# Use of and short-term impacts of new cycling infrastructure in inner-Sydney, Australia: a quasi-experimental design

**DOI:** 10.1186/s12966-015-0294-1

**Published:** 2015-10-06

**Authors:** Chris Rissel, Stephen Greaves, Li Ming Wen, Melanie Crane, Chris Standen

**Affiliations:** Sydney School of Public Health, University of Sydney, Sydney, Australia; Institute of Transport and Logistics Studies, University of Sydney, Sydney, Australia; Sydney School of Public Health, University of Sydney and Sydney Local Health District, Sydney, Australia

**Keywords:** Bicycling, Infrastructure, Environment

## Abstract

**Background:**

Given increasing investment in new cycling infrastructure, it is important to understand its impacts. The Sydney Transport and Health Study evaluates a new 2.4 km bi-directional separated bicycle path in inner-Sydney. This paper describes the users of the new bicycle path, and examines its short-term impacts upon cycling behaviour and perceptions of the local environment.

**Methods:**

Data were collected from two bike counts at two intersections on the new bicycle path in the intervention area in 2013 and 2014. On-line surveys collected individual participant data in the intervention area and a similar comparison area before the bicycle path was built (2013), and 12 months later (four months after completion) (*n* = 512). The data included self-reported cycling behaviour, use of the new bicycle path and perceptions of changes in the local environment.

**Results:**

Bike counts at two sites on the new bicycle path reported an increase of 23 % and 97 % respectively at 12 months. However, among the participants in the cohort, there was no change in the self-reported weekly frequency of cycling. One in six (approximately 15 %) participants reported using the new bicycle path, with most users (76 %) living in the intervention area. Bicycle path users were most likely to be frequent riders (at least weekly) [adjusted odds ratio (AOR) = 7.50, 95 % CI 3.93–14.31], be a high intensity recreational rider (AOR = 4.38, 95 % CI 1.53–12.54) or a low intensity transport rider (AOR = 2.42, 95 % CI 1.17–5.04) and live closer to the bicycle path (AOR = 1.24, 1.13–1.37). Perceptions that the neighbourhood was more pleasant, that there were more people walking and cycling were significantly higher in the intervention area at 12 months (both *P* values <0.05).

**Conclusions:**

Existing cycling behaviour and proximity to the bicycle path were associated with the use of the new bicycle path. Increased use of the new bicycle path as reported by the participants in the intervention area and increased cycling recorded by the bike counts may be due to existing cyclists changing routes to use the new path, and more cyclists from outside the study area using the new path, as study participants did not increase their frequency of cycling. Increases in cycling frequency in the intervention neighbourhood may require a longer lead time, additional promotional activities and further maturation of the Sydney bicycle path network.

**Key message:**

Understanding how new cycling infrastructure impacts communities can influence the promotion of such infrastructure.

## Background

The many health [1, 2] and environmental benefits [3] of cycling are increasingly being recognised in national or regional cycling policies and plans [4–8]. Strategies to increase levels of cycling include new cycling specific infrastructure, such as separated bicycle paths, speed reduction policies and promotional campaigns including driver education [9, 10]. There is also wider public support for investment in active travel infrastructure [11].

Qualitative research with occasional or non-cyclists consistently finds that safety concerns are a dominant reason given for why people do not use a bicycle for trips that could be cycled [12]. A consistent recommendation in many policy documents to support non-cyclists to begin cycling is to provide bicycle paths separated from motor vehicles [13]. Cities and countries with high bicycle mode share generally have more developed cycling infrastructure [14, 15].

Given the demand for and increasing investment in cycling infrastructure in some regions [8, 16], it is important that the impacts of new infrastructure be comprehensively evaluated. However, building new infrastructure can be expensive and takes time, and it is not possible to evaluate these initiatives applying experimental research designs commonly used in medical research where individuals are randomised to different treatments. Quite reasonably, much transport research involves natural experiments [17] with before-and-after designs using repeated observations with population level data [18].

One recent evaluation of new high-quality, traffic-free routes for walking and cycling with a baseline and follow-up study found living nearer the infrastructure predicted increases in physical activity two years later relative to those living farther away, and the effects were larger among participants without a car [19]. Another study of the promotion of cycling infrastructure in Australia found while there was increased use of the infrastructure, there were no increases in cycling participation compared with a similar area that did not receive promotional communications [20].

A ‘natural experiment’ in the UK iConnect study has provided good opportunities to examine the impacts of new walking and cycling infrastructure on usage and health benefits [21, 22], and sometimes with unexpected or mixed results [23]. Overall, well designed and connected infrastructure does tend to lead to increased use and physical activity [19, 24, 25] with closer distance to the infrastructure being an important factor [26, 27].

In Sydney, Australia, an opportunity arose to evaluate new cycling infrastructure being built by the City of Sydney as part of its expanding bicycle network [13]. A partnership of key agencies was able to attract research funding to examine health and transport outcomes 12 and 24 months after a new 2.4 km bicycle path was built, and also to compare these with a comparable inner city area [28]. This paper assesses the short term impact of the new cycling infrastructure on awareness and use of the new infrastructure and addresses the research question of what changes in cycling behavior and perceptions of the neighbourhood were observed.

## Methods

### Design

This study uses a longitudinal, quasi-experimental design in which a panel of participants were recruited from an intervention and comparison area and surveyed before and after construction of a new bicycle path. Baseline data (before construction of the bicycle path) were collected on-line in September–October 2013, with a questionnaire component and an on-line 7 day travel diary (diary data not reported here). Participants were recruited through various methods (online consumer panel, cold calling, social media, electronic circulation lists, mailbox drops and intercept events focused around cycling) into the panel with agreeable participants then sent a URL to begin the survey. The 12-month data were collected in September–October 2014 (four months after completion of the bicycle path) using the same methods, with some additional question items to examine awareness of and use of the new bicycle path.

### Setting

**Fig. 1:**
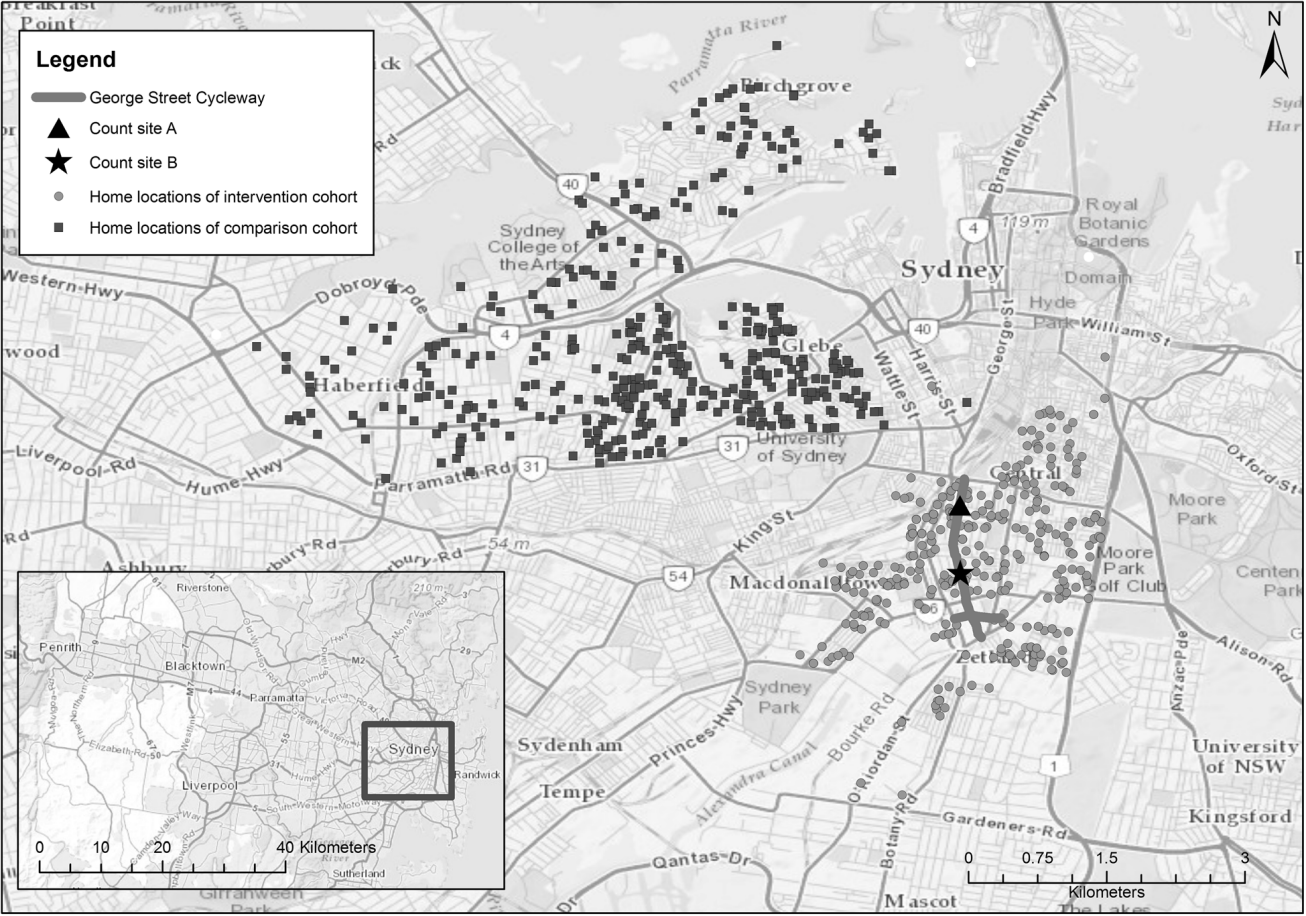
Location of study participants in intervention and comparison area and new bicycle path

Participants were identified as living in either the intervention or comparison area, defined in the intervention area by postcodes in close proximity to the new bicycle path and living not more than 2.5 km away. The comparison area included neighbourhoods a similar distance from the central business district and with a similar demographic profile, and where the local council had no plans to modify infrastructure during the study period (see Fig. 1). However, because distance to cycling infrastructure is potentially an important variable, we further defined exposure as the network distance between the respondent’s residential address and closest point of the new bicycle path. Unprompted and prompted awareness of the new bicycle path, usage and intention to use the bicycle path were asked at follow-up.

### Participants

Participants were eligible subject to the geographic constraints described above if they were aged 18–55 years, had ridden a bicycle in their life and had no current disability preventing them from riding, and had sufficient English to complete the survey.

#### Travel behaviour

Participants were asked about their cycling behaviour including bicycle ownership ‘Is there a bicycle at your home that is available for you to ride?’ with yes/no options [29], frequency of cycling ‘How often do you usually travel by bicycle (for any purpose)?’ with the response options including seven categories ranging from ‘never’, ‘less than once a month’, ‘1–2 days a month’, ‘1–2 days a week’, ‘3–4 days a week’, ‘5–6 days a week’ and ‘everyday’ [29], and usual travel to work or study ‘What is the main way you travel to/from work or study?’ with the response options being public transport, motor vehicle, bicycle, walking or no travel) [30]. General baseline cycling intensity and purpose was determined by self-perceptions of being a low or high intensity recreational or transport rider [31].

#### Neighbourhood perceptions

Questions on changes to the neighbourhood environment relative to 12 months previously were asked at follow-up, with response options agreement on a five point scale (strongly agree to strongly disagree) with the following statements:

- ‘Compared with 12 months ago, I feel more connected with my neighbours’
- ‘Compared with 12 months ago, my neighbourhood is more pleasant’
- ‘Compared with 12 months ago, there are more people walking in my local area’
- ‘Compared with 12 months ago, there are more people cycling in my local area’

Responses were dichotomised to ‘strongly agree/agree’ and ‘strongly disagree/disagree/not sure’.

#### Demographic and socioeconomic factors

Demographic correlates with potential variation in reporting behaviour included sex and age. Given the high levels of education and income, education was dichotomised into tertiary or less than tertiary level, and annual household income was categorised in intervals from less than $20,000 to over $140,000 and dichotomised at AU$80,000+ or less. Variables were dichotomised because of concerns about multiple categories reducing statistical power.

#### Bike counts

The City of Sydney local council conducted counts of cyclists at 100 intersections across the City in October 2013 and October 2014, at the end of data collection for this study. The bicycle path was completed in June 2014. One site (A) was at the northern end (along the roadway where the new bicycle path being evaluated was built, and another half way along the path (site B) – see Fig. 1. Counts recorded any cyclist moving in any direction at the intersection, and were conducted for 3 h in the morning and afternoons to cover peak periods, from 6–9 am and 4–7 pm, at each intersection.

#### Analysis

Analyses were performed using Stata 13 (StataCorp, LLC, College Station, TX). Characteristics of the baseline and cohort samplers were compared using Chi Square tests. Sample characteristics of the cohort were compared using simple McNemar and ANOVA tests for >2 categories. Changes in cycling behaviour over time were investigated using mixed-effects logistic regression models. Logistic regression analyses were used to examine univariate differences between how participants in the intervention and comparison neighbourhoods interacted with the bicycle path (awareness, use, future intention to use), neighbourhood perceptions and demographic characteristics (age, sex, education, income) (see Table 2). In the multivariate model, awareness, use and future intention to use were modelled adjusting for age, sex, education and income. Logistic regression analyses were also used to determine predictive characteristics of bicycle path users, including cycling frequency, purpose and residential distance from the bicycle path (see Table 3). Distance from residential address to the bicycle path was rescaled to intervals of 500 m, then 100 m. To determine distance from the bicycle path, the bicycle path was coded to zero and every increment further from the bicycle path recoded as a negative value.

The research has been approved by the Human Research Ethics Committee, The University of Sydney (protocol number 2012/2411).

## Results

### Response rates

A total sample of 846 questionnaire responses (398 in the intervention and 448 in the comparison area) was collected at baseline. The 675 participants who agreed to continue in the study, remained living in the intervention or comparison area and provided complete data for the questionnaire and/or diary were invited to participate in the follow-up questionnaire 12 months later, of which 512 participants agreed (75.9 %). The characteristics of participants at baseline and follow-up are shown in Table 1. Those participants who were retained in the study, were more likely to be older and earn a high income, and less likely to cycle regularly or cycle to work than the baseline sample.

**Table 1:** Characteristics of participants and self-reported cycling behaviours at baseline and 12-month follow-up

	Baseline	Follow-up	Cohort comparison *P*
	*n* = 846	*n* = 512	
Bicycle ownership			
No	294 (34.7)	179 (34.9)	0.50
Yes	552 (65.3)	334 (65.1)	
Cycling frequency			
At least weekly	237 (28.1)	125 (24.4)	<0.001
Within 12 months	236 (27.9)	150 (29.3)	
Longer than a year	373 (44.1)	237 (46.3)	
Usual mode to work			
Bicycle	113 (13.4)	48 (9.4)	<0.001
Walk	168 (19.9)	101 (19.7)	
Public transport	332 (39.2)	194 (37.9)	
Car	198 (23.4)	130 (25.4)	
No travel	35 (4.1)	39 (7.6)	
Age			
18–24	149 (17.6)	70 (13.7)	<0.001
25–34	214 (25.3)	111 (21.7)	
35–44	217 (25.7)	141 (27.5)	
45–55	266 (31.4)	190 (37.1)	
Sex			
Male	494 (41.9)	202 (39.5)	0.12
Female	352 (58.1)	310 (62.5)	
Education			
Less than tertiary	255 (30.4)	131 (25.6)	0.10
Tertiary or higher	585 (69.6)	380 (74.4)	
Income			
Less than $80 K (AUS)	286 (37.3)	136 (30.0)	0.03
$80 K or more	481 (62.7)	318 (70.0)	

### Bike counts

In October 2013, 812 cyclists were counted at Site A, and 1001 cyclists in October 2014, a 23 % increase. At Site B, 201 cyclists were counted in October 2013 and 395 in October 2014, a 97 % increase. The change in rates of cycling over these 12 months across the whole of the City of Sydney was a 3 % increase.

### Cycling behaviour

Weekly frequency of cycling six months following completion of the bicycle path remained higher in the intervention (29.2–25.8 % at follow-up) area than the comparison area (22.4–23.2 % at follow-up) (*p* = 0.04), and this did not change over time (*p* = 0.2). There was a reduction in travel to work or study by bicycle (*p* = 0.001) between baseline and follow-up, observed across both intervention (14.1–11.3 % at follow-up) and comparison areas (12.7–7.7 % at follow-up) (*p* = 0.40). There was no change in bicycle ownership, and there were no significant changes between baseline and follow-up in the intervention and comparison areas in the reported ease of cycling or perception of bicycle facilities in their local area. Those more likely to perceive there were bicycle facilities in their local area were more likely to be residents in closer proximity to the bicycle path with an odds ratio of 1.11 (95 % CI 1.04–1.19).

Awareness of the bicycle path, use and intention to use the path was significantly higher in the intervention area compared with the comparison area at follow-up (see Table 2). Seventy five percent of bicycle path users lived in the intervention area. Three times as many participants in the intervention area were aware of the new path (60 %) compared with the comparison area (19 %) (Adjusted Odds Ratio (AOR) = 5.99, 95 % Confidence Interval (CI) 3.87–9.27). Use of the bicycle path was significantly higher by those residing in the intervention area (24 %) compared with the comparison area (7 %) (AOR = 3.58, 95 % CI 2.01–6.40). Intention to use the bicycle path was more than double by intervention area residents (36 %) compared with comparison area residents (16 %) (AOR = 2.77, 95 % CI 1.76–4.37).

**Table 2:** Comparison between intervention and comparison neighbourhoods of awareness of, use, intention to use a new bicycle path, weekly cycling frequency and neighbourhood factors at follow-up (*n* = 512)

	Comparison Area *n* = 272 (%)	Intervention Area *n* = 240 (%)	Odds ratio	Adjusted odds ratio (95 % CI)^a^	*P* value
Bicycle path interaction at follow-up					
Awareness	18.8	60.0	6.49	**5.99 (3.87–9.27)**	**<0.001**
Use of bicycle path	7.0	23.8	4.15	**3.58 (2.01–6.40)**	**0.001**
Intention to use					
(Very likely/likely)	15.8	35.8	2.97	**2.77 (1.76–4.37)**	**<0.001**
Neighbourhood factors					
Compared with 12 months ago (agree/strongly agree):					
I feel more connected with my neighbours	40.2	37.6	0.88	1.09 (0.72–1.58)	0.612
My neighbourhood is more pleasant	29.5	47.5	2.14	**2.44 (1.63–3.66)**	**<0.001**
There are more people walking in my local area	37.6	53.7	1.94	**2.04 (1.37–3.03)**	**<0.001**
There are more people cycling in my local area	58.7	74.8	2.04	**2.48 (1.62–3.79)**	**<0.001**
Agree/strongly agree that:					
It is easy to ride a bicycle around your local area	64.0	71.3	1.39	1.37 (0.90–2.08)	0.201
There are bicycle facilities in my local area	74.6	85.4	2.12	**2.08 (1.26–3.42)**	**<0.001**
Cycling frequency					
Bicycled in past week	23.2	25.8	1.16	1.07 (0.67–1.69)	0.767

^a^Adjusts for age, sex, income and education

Bold text highlights statistically signifcant associations

Participants in the intervention area were significantly more likely to agree/strongly agree that compared to 12 months ago their neighbourhood was more pleasant than participants in the comparison area (48 % Vs 30 %) (AOR = 2.44, 95 % CI 1.63–3.66) that there were more people walking (54 % Vs 38 %) (AOR = 2.04, 95 % CI 1.37–3.03) and more people cycling (75 % Vs 59 %) (AOR = 2.48, 95 % CI 1.62–3.79) in their local area (Table 2). There was no significant difference in participants reporting that they felt more connected to their neighbours. The associations between these outcomes and by intervention area were confirmed when analyses were conducted by distance of residence to the nearest point of the bicycle path as the exposure measure (data not shown).

Across both the intervention and comparison areas, one in six (approximately 15 %) residents reported they had used the new bicycle path, with most users living in the intervention area (76 %). Bicycle path users were most likely to be a high intensity recreational rider (AOR = 4.38, 95 % CI 1.53–12.59) or a low intensity transport rider (AOR = 2.42, 95 % CI 1.17–5.04) and had ridden their bicycle in the past week (AOR = 7.50, 95 % CI 3.93–14.31) (see Table 3). As distance from the bicycle path decreased (500 m increments), likelihood of using the bicycle path significantly increased (AOR = 1.24, 95 % CI 1.13–1.37).

**Table 3:** Characteristics of new bicycle path users and factors associated with new users

	%	Odds Ratio (95 % CI)	Adjusted Odds Ratio 95 % CI^a^	*P* value
Proportion had used cycleway (*n* = 75)	14.7			
Age				
18–24	15.5	1.0	1.0	
25–34	20.9	1.44 (0.62–3.36)	0.54 (0.18–1.57)	0.890
35–44	18.4	1.23 (0.53–2.85)	0.73 (0.25–2.15)	0.953
45–55	9.6	0.58 (0.25–1.34)	0.42 (0.14–1.24)	0.192
Sex				
Female	13.9	1.0	1.0	
Male	16.3	1.21 (0.74–1.99)	0.64 (0.34–1.21)	0.306
Education				
Less than tertiary	13.0	1.0	1.0	
Tertiary or higher	15.5	1.23 (0.69–2.20)	0.83 (0.39–1.77)	0.908
Income				
Less than $80 K (AUS)	13.2	1.0	1.0	
$80 K or more	17.0	1.34 (0.75–2.39)	1.26 (0.63–2.54)	0.551
Weekly cycling frequency				
Less than weekly		1.0	1.0	
At least weekly		7.44 (4.41–12.56)	**7.50 (3.93–14.31**)	**<0.001**
Cyclist type				
Low intensity recreational	7.0	1.0	1.0	
High intensity recreational	30.3	5.79 (2.45–13.68)	**4.38 (1.53–12.59)**	**0.026**
Low intensity transport	25.4	4.54 (2.50–8.22)	**2.42 (1.17–5.04)**	**0.032**
High intensity transport	31.0	5.97 (2.72–13.09)	2.40 (0.90–6.44)	0.598
Distance from bicycle path in 500 m^b^		1.21 (1.12–1.31)	**1.24 (1.13–1.37)**	**<0.001**
Distance from bicycle path in 100 m^b^		1.04 (1.02–1.05)	**1.04 (1.02–1.06)**	**<0.001**

^a^Adjusted for all other variables in the model

^b^One or the other included in the model at one time

Bold text highlights statistically signficant associations

## Discussion

Since the completion of the new bicycle path, participants in the intervention area reported significantly greater awareness, use and intention to use the path compared with comparison area participants. There were also higher levels of agreement that there were more people cycling and walking in the intervention area, and that the neighbourhood was more pleasant. Proximity to the cycle path was significantly associated with these benefits. The bicycle counts confirm that there was an increase in users of the new bicycle path. However, there was no increase in the frequency of having cycled in the past week among the cohort of participants, nor any change in the proportion usually travelling to work/study by bicycle.

The observed increase in cycling may be due to some funnelling of existing riders to the new path (a form of induced travel), or riders outside the study areas cycling through the intervention area on the new bicycle path. To increase cycling frequency or bicycle travel mode, sustained promotional programs may be needed over time. Further improvements to the City of Sydney bicycle network to increase connectivity may also make cycling more attractive [32].

The pattern of cycling in the Greater Metropolitan Sydney region (using census journey to work data from 2001, 2006, 2011) has been that up to 2011, overall across Sydney there was little change in bicycle mode, but this masked decreases in bicycle commuting in outer Sydney and marked increases in the inner city [33, 34]. It is also possible that because at baseline there were already quite high levels of cycling among study participants, a local ceiling may have been reached in the short term.

Cycling infrastructure such as bike lanes, especially bike paths separated from motor traffic, have consistently been associated with higher levels of cycling. This has been shown at cross sectional and ecological levels [15, 35–37], and increasingly from prospective quasi experimental trials [38–40]. Users of the new bicycle path were more likely to be existing riders (high intensity recreational or low intensity transport cyclists) and lived closer to the bicycle path. Demographic factors were not significantly associated with path use.

A number of limitations of the analysis should be noted. Bike count data were of all cyclists through the intersection, not only those cycling along the new bicycle path. Some of the increased cycle traffic may be existing cyclists changing routes to use the new facility. Biases introduced through recruitment of younger participants who have ridden a bicycle in their life may have meant the sample is less representative of the inner Sydney population, or introduced other biases. Loss to follow-up at 12 months reduces the power of the analysis, but the sample was determined by expected change in the primary outcomes of cycling behavior at the longer term follow-up, and so should be sufficient at the short term follow-up point. The neighbourhood perception questions had not been validated. Because there was no increase in the number of participants cycling at this time, prospective analyses examining factors associated with new cyclists were not possible.

## Conclusions

Existing cycling behaviour and proximity to the bicycle path were the main factors associated with the use of the new bicycle path. Increased use of the new bicycle path as reported by the participants in the intervention area and increased cycling recorded by the bike counts may be due to existing cyclists changing routes to use the new path, and more cyclists from outside the study area using the new path, as study participants did not increase their frequency of cycling. Increases in cycling frequency in the intervention neighbourhood may require a longer lead time, additional promotional activities or further maturation of the Sydney bicycle path network.
